# Adsorption of Rhodamine B from Simulated Waste Water onto Kaolin-Bentonite Composites

**DOI:** 10.3390/ma15124058

**Published:** 2022-06-07

**Authors:** Haijie He, Kuan Chai, Tao Wu, Zhanhong Qiu, Shifang Wang, Jie Hong

**Affiliations:** 1 College of Civil and Architectural Engineering, Zhejiang University, Hangzhou 310000, China; he_haijie@zju.edu.cn; 2 College of Civil and Architectural Engineering, Taizhou University, Taizhou 318000, China; chaikuan1996@163.com (K.C.); hongjieziliao@126.com (J.H.); 3 Fangyuan Construction Group Co., Ltd., Taizhou 317700, China; 4 School of Civil Engineering, Shenyang Jianzhu University, Shenyang 110000, China; 5 College of Civil Engineering and Architecture, Jiangsu University of Science and Technology, Zhenjiang 212000, China; 6 Jiangsu Province Engineering Research Center of Geoenvironmental Disaster Prevention and Remediation, Jiangsu University of Science and Technology, Zhenjiang 212000, China; 7 School of Mechanics and Civil Engineering, China University of Mining and Technology, Xuzhou 221018, China

**Keywords:** dye, characterization, adsorption, kinetics, isotherm

## Abstract

Organic dye rhodamine B is one of the common organic pollutants in the water and soil environment. This study investigated the feasibility of removing rhodamine B from an aqueous solution through adsorption by kaolin, kaolin-sodium bentonite, and kaolin-organic bentonite. Batch adsorption test results showed that the maximum adsorption quantities of kaolin, kaolin-sodium bentonite, and kaolin-organic bentonite were 7.76 mg/g, 11.26 mg/g, and 12.68 mg/g, respectively, implying that the addition of bentonite to kaolin can effectively improve its adsorption capacity for rhodamine B. Moreover, the Langmuir isotherm model is suitable to describe the adsorption of rhodamine B by kaolin and kaolin-sodium bentonite, while it is preferable to use the Freundlich isotherm model in the case of kaolin-organic bentonite. The adsorption kinetic characteristics of rhodamine B, by these three adsorbents, are suitable to be described with a pseudo-second order kinetic model. Furthermore, the characteristics of the above three adsorbents were characterized by scanning electron microscopy (SEM), X-ray diffraction (XRD), and Fourier transform infrared spectroscopy (FTIR). The above results indicated that kaolin and organic bentonite can be used to design efficient adsorbents for organic pollutants similar to rhodamine B.

## 1. Introduction

Dyes are widely used in industries such as textiles, printing and dyeing, tanning, and food. Dye wastewater is generated in the process of dye production and use, resulting in environmental pollution [1,2,3]. Dye wastewater generally has the characteristics of poor biodegradability, a wide range of acidity and alkalinity, high toxicity, large chromaticity, and complex composition, which not only affects the aesthetics of the environment but also does harm to human body, animals, and ecology [4,5]. Rhodamine B is a kind of triphenylmethane alkaline water-soluble organic dye with fresh peach color, which is used in the textile industry, colored glass, and other industries. However, being potentially toxic to the human body, it can cause eye burns and stimulate the skin, respiratory tract, and gastrointestinal tract; thus, it is listed as Class 3 carcinogen by the International Agency for Research on Cancer of the World Health Organization [6,7]. When rhodamine B is discharged into the environment and consumed by humans and animals, it causes serious pollution and great damage. Therefore, it is crucial to treat the dye wastewater before it is discharged into the receiving water body, which is of great significance to maintain the ecological environment [8].

Many physical, chemical, and biological methods have been proposed to treat dye wastewater, mainly including flocculation, adsorption, membrane separation, electrochemistry, advanced oxidation, and biological treatment [9,10]. Dyes could be removed by microbial adsorption [11,12]. However, current technologies limit their large-scale application due to some economic and environmental shortcomings. For example, the electrochemical method consumes a lot of power and money [13], the membrane separation method has the problem of severe membrane blockage and high cost [14], and the advanced oxidation method forms toxic by-products and is costly [15], while the biological treatment method has poor adaptability and long treatment time [16]. Compared with these mentioned methods, the adsorption method has attracted extensive attention because of its economic feasibility, environmental friendliness, and operation simplicity [17].

Adsorption is considered to be an efficient physicochemical separation method for purifying wastewater, and it is considered to be the most commonly used physicochemical method [7,18]. The adsorption process is closely related to the type and physicochemical properties of adsorbents, especially the organic and inorganic properties of the materials used [19]. In addition, the cost of the adsorption decolorization process mainly depends on the adsorbent cost and the regeneration cost of the adsorbent [20]. Activated carbon has advantages, including a large specific surface area and strong adsorption capacity, making it the most widely used adsorbent at present [21]. However, the high cost and difficult regeneration of activated carbon limit its application, leading to the development of alternative adsorbents, which are cheaper and easy to regenerate. Clay minerals, such as kaolinite and montmorillonite, have been proven to be cost-effective and efficient dye adsorbents [22,23].

Clay has the advantages of high specific surface area, porosity, cation exchange capacity, accessibility, and adsorption performance, and it is a promising adsorption material [18,24]. Kaolin and bentonite are common adsorption materials; the former is mainly composed of kaolinite, illite, and a small amount of montmorillonite, while the latter is mainly composed of montmorillonite. In this paper, rhodamine B solution was selected to simulate dye wastewater, and the removal efficiency for rhodamine B from an aqueous solution by kaolin, kaolin-sodium bentonite, and kaolin-organic bentonite was studied. A series of experiments were performed to study the effects of several parameters on the adsorption capacity, including the initial concentration of rhodamine B solution, temperature, pH, and the kaolin-organic bentonite dosage. Furthermore, the adsorption kinetics and adsorption isotherms were explored.

## 2. Materials and Methods

### 2.1. Preparation of Adsorbents

In this paper, sodium bentonite and kaolin were used as adsorbents, and all the soils used were purchased from Hebei Lingshou Xingyuan Mineral Company (Shijiazhuang). KOH and HCl solution, purchased from Shanghai Anpu Experimental Technology Co., Ltd. (Shanghai, China), were used as reference materials for titration solution to adjust the pH of contaminated solution. Cetyltrimethylammonium bromide (CTMAB) and rhodamine B, used in the test, were of analytical pure and purchased from Shanghai Aladdin Biochemical Technology Co., Ltd (Shanghai, China).

The organic bentonite used in this paper was CTMAB-bentonite prepared by the wet method with CTMAB as the surfactant [25]. Firstly, 100 g of sodium bentonite was weighed and put into 1 L beaker, and 500 mL of deionized water was added to prepare 5% slurry; 25 g of CTMAB was weighed, which was then added into the slurry, and the solution was stirred evenly. After shaking in a constant temperature shaker, at 70 °C for 2 h, it was aged overnight in an electric blast drying oven at 70 °C until the cation surfactant was completely exchanged with the cation in sodium bentonite. Then, the prepared sample was taken out, suction filtrated, and washed. We detected the bromine ion of the filtrate with a silver nitrate solution. When no bromine ion was detected, it proved the complete replacement. Subsequently, the organic bentonite was put into a 100 °C electric blast drying oven. After drying, the organic bentonite was taken out for grinding and passed through a 100-mesh sieve. Thus, the preparation of CTMAB-bentonite was completed.

The three adsorbents used in this test were kaolin, kaolin-sodium bentonite (mass ratio 9:1), and kaolin-organic bentonite (mass ratio 9:1). In order to make kaolin-organic bentonite adsorbent, 5 g of CTMAB-bentonite was mixed with 45 g of kaolin and 500 mL of deionized water. These materials were stirred evenly, and they were vibrated for 2 h, in a constant temperature water bath shaker, at 70 °C. Then, the obtained mixture was put into an electronic blast drying oven, and it was dried at 105 °C for 24 h. After taking out the dried sample and grinding, it passed through a 60-mesh sieve to obtain kaolin-organic bentonite adsorbent. The preparation method of kaolin-sodium bentonite was almost the same as that of kaolin-organic bentonite; the CTMAB-bentonite, in the above method, was replaced with sodium bentonite.

### 2.2. Characterization of Adsorbents

The three adsorbents passed through a 200-mesh sieve to obtain a sample with a particle size of 0.074 mm. The surface morphology, X-ray diffraction (XRD) patterns, and structural composition of three adsorbents were studied by SEM (S-4800, Hitachi, Tokyo, Japan), X-ray diffractometer (D8 ADVANCE, Brooke, Karlsruhe, Germany) and Fourier infrared spectrometer (NICOLET-5700, Thermoelectric Company, Waltham, MA, USA). The infrared spectra (4000–400 cm^−1^) was measured using the KBr pressed disk technique.

### 2.3. Batch Adsorption Test

In order to study the adsorption efficiency of the three adsorbents (kaolin, kaolin-sodium bentonite, and kaolin-organic bentonite) on rhodamine B simulated wastewater, the effects of the initial concentration of rhodamine B solution, adsorption time, and temperature, on the removal of rhodamine B, were studied through batch adsorption tests. Additionally, we explored whether the pH value and adsorbent dosage influenced the removal of rhodamine B by kaolin-organic bentonite. The specific test scheme is shown in Table 1.

Firstly, the adsorbent of the target mass was put into a 50 mL centrifuge tube, and then, 10 mL of rhodamine B solution of the target concentration was added. We placed the centrifuge tube into a constant temperature shaker and vibrated it with a speed of 300 rpm for the corresponding time under target temperature and pH value. The pH was controlled by a bench pH meter, NaOH, and HCl solution. Then, the sample was taken out and put into a high-speed centrifuge, which separated the sample at 4000 rpm to stop the adsorption. After centrifugation for different target times, 1 mL of supernatant was taken. We diluted it 20 times, measured its absorbance at 554 nm wavelength using the T6 ultraviolet spectrophotometer (Beijing Persee General Instrument Co., Ltd., Beijing, China), and determined the concentration of rhodamine B, according to the calibration curve of rhodamine B (linear correlation coefficient R^2^ = 0.9995). Absorption spectra were recorded using the ultraviolet spectrophotometer in a spectrometric quartz cuvette. Each adsorption test was repeated three times, and the average value was recorded.

The removal rate and adsorption amount for rhodamine B, by each adsorbent in the test, were calculated according to Equations (1) and (2):

(1) $$\mathrm{Removal}\;\mathrm{rate}:\;R=\frac{c_{0}-c}{c_{0}}\times 100\%$$

(2) $$\mathrm{Adsorption}\;\mathrm{amount}:\;q=\frac{(c_{0}-c)\times V}{m}$$

where *q* is the adsorption amount (mg/g); *c*_0_ is the initial concentration of rhodamine B solution (mg/L); *c* is the residual concentration of rhodamine B solution after adsorption (mg/L); *V* is the volume of simulated wastewater (L); *m* is the adsorbent dosage (g).

## 3. Results and Discussion

### 3.1. Characterization of Adsorbents

**Figure 1:**
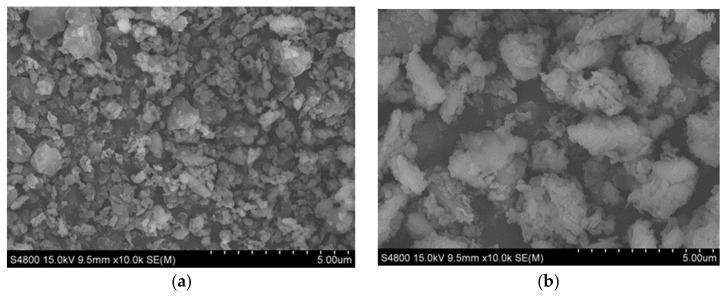

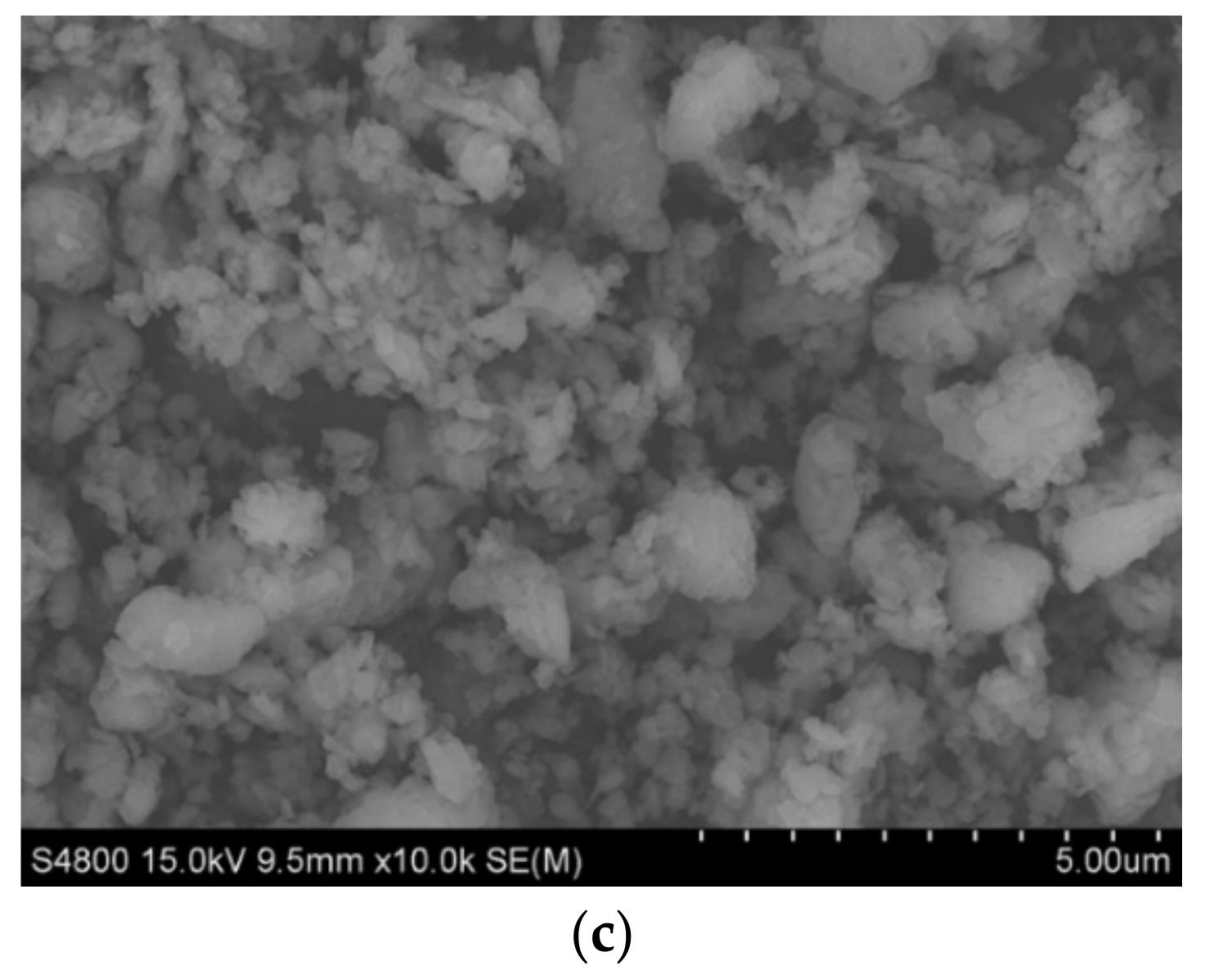
SEM images of different adsorbents: (**a**) kaolin; (**b**) kaolin-sodium bentonite; (**c**) kaolin-organic bentonite.

Figure 1a–c show the SEM images of kaolin, kaolin-sodium bentonite, and kaolin-organic bentonite, respectively, displaying the surface morphology and microstructure of the three adsorbents. It can be seen, from Figure 1a, that kaolin has small particles and a loose structure, and the particle size of kaolin particles is mainly distributed in around 3 μm. With the addition of sodium bentonite (Figure 1b), the particles of kaolin-sodium bentonite agglomerate and the particle size of kaolin-sodium bentonite increases to about 15 μm [26,27]. This is because, with the increase in bentonite content, the cementation coefficient of kaolin-sodium bentonite is also increasing, which promotes the formation of agglomerates. After adding organic bentonite to kaolin (Figure 1c), some polymers are also formed, but its effect on soil particle agglomeration is lower than that of sodium bentonite. This may be because the addition of the CTMAB modifier expands the layer spacing of bentonite, promotes the formation of stripping bentonite, and reduces the cementation degree of kaolin. In general, after adding sodium bentonite or organic bentonite to kaolin, the soil particles increase in size. This phenomenon is mainly due to the increased cementing property between soil particles.

**Figure 2:**
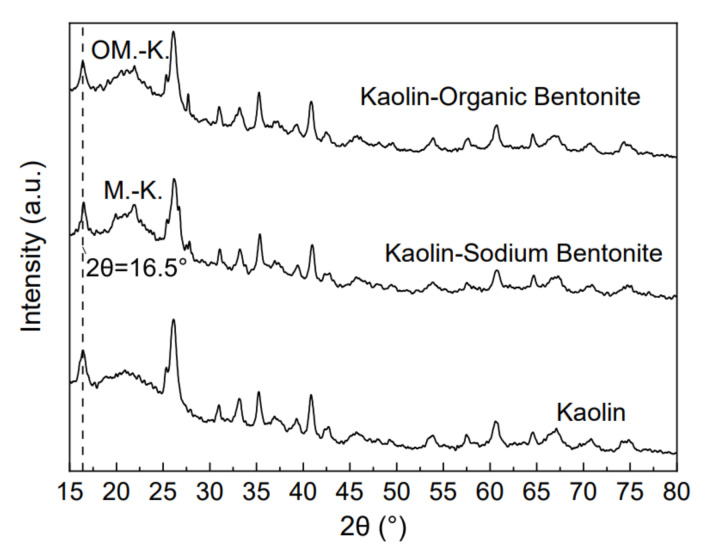
XRD patterns of the three adsorbents.

XRD analysis can reflect the changes of material composition and spatial structure. The XRD patterns of kaolin, kaolin-sodium bentonite, and kaolin-organic bentonite are shown in Figure 2. As shown in the figure, the layer spacing of the three adsorbents is 0.60 nm, and the value of 2θ, corresponding to the d_001_ characteristic peak, is about 16.5°. From the figure, it can be seen that the characteristic peak positions of the three adsorbents are basically the same; this is because there is no obvious change in kaolinite layer spacing after the introduction of sodium bentonite and organic bentonite. However, when 2θ is 22°, the characteristic peak values of kaolin-sodium bentonite and kaolin-organic bentonite adsorbents increase sharply, as shown at the M.-K. position in the figure. This is because the addition of sodium bentonite makes the sodium bentonite bond with kaolin to produce a new kaolin bentonite polymer; it is a new characteristic peak formed by this polymer. The characteristic peak value at the OM.-K. position is reduced because the introduction of the modifier reduces the cementation degree between kaolin and sodium bentonite, and it destroys the polymer structure. This phenomenon can also be reflected in Figure 1. The position and increased amplitude of other peaks are basically the same as that of kaolin. XRD results show that the introduction of sodium bentonite increases the montmorillonite content, internal space, adsorption points, and thus, the adsorption capacity. Montmorillonite has strong adsorption advantages due to its large natural specific surface area, and the introduction of montmorillonite enhances the adsorbent’s adsorption capacity.

**Figure 3:**
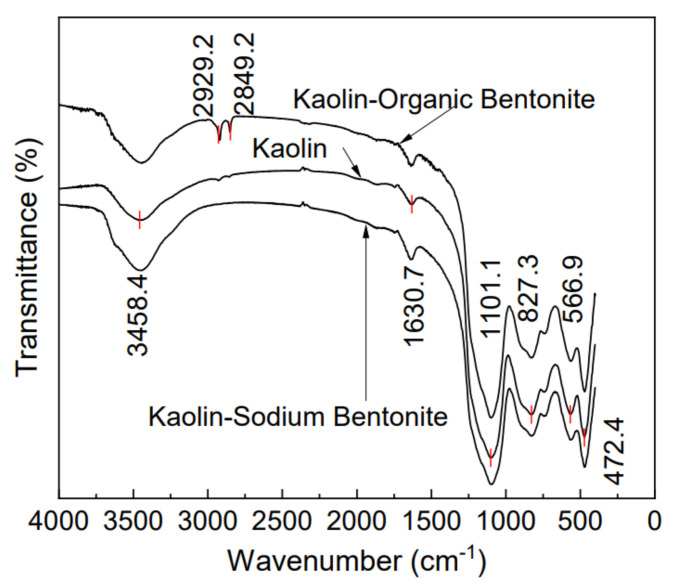
FTIR spectra of the three adsorbents.

FTIR spectra of kaolin, kaolin-sodium bentonite, and kaolin-organic bentonite are shown in Figure 3. It can be seen, from the figure, that the FTIR spectra of the three adsorbents have roughly the same peak shape, indicating that the addition of a small amount of sodium bentonite or organic bentonite does not significantly change the basic skeleton of kaolin. The characteristic peak 3458 cm^−1^ is caused by the stretching vibration of H-O-H, and the characteristic peak 1630 cm^−1^ is caused by the left and right bending vibration of H-O-H [28]. The characteristic peaks 2929 cm^−1^ and 2849 cm^−1^ are caused by the introduction of -CH_3_ in the CTMAB modifier, and the track between the characteristic peak 471–1101 cm^−1^ is related to the stretching and bending vibration of Si-O-Si and Si-O-Al [23]. On the whole, the transmittance of kaolin is the lowest, and the transmittance is improved after the introduction of sodium bentonite and organic bentonite.

### 3.2. Effect of Adsorption Time on Removal of Rhodamine B by Different Adsorbents

**Figure 4:**
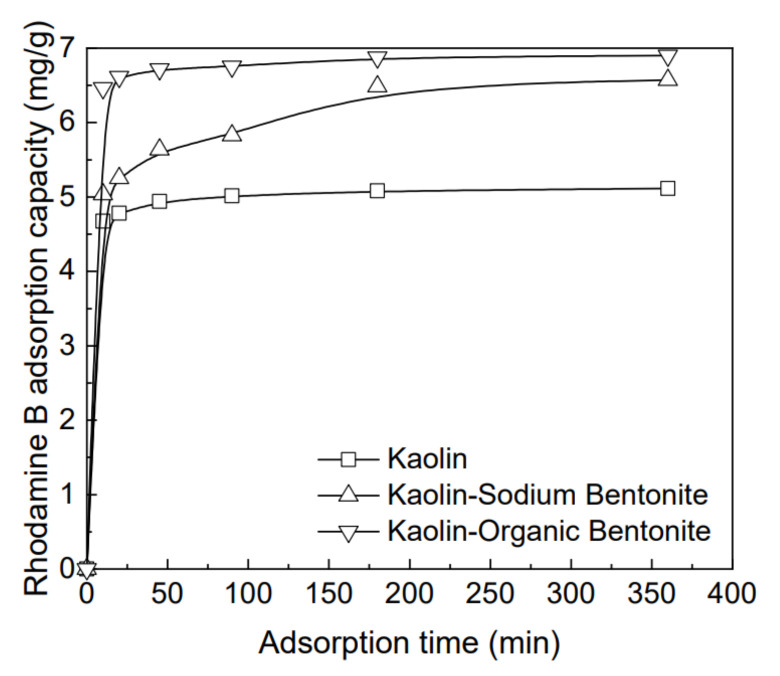
Variation of adsorption amounts of different adsorbents for the rhodamine B solution, with an initial concentration of 200 mg/L versus adsorption time.

Under the conditions of pH 9.1 and temperature 25 °C, the adsorption process of the three adsorbents for rhodamine B solution, with an initial concentration of 200 mg/L, is chosen as the representative result (Figure 4). It is obvious that adsorption time has a significant effect on the removal quantity of rhodamine B. In the first 10 min, the adsorption amount for rhodamine B by each adsorbent increases rapidly with the increase in adsorption time, and then, with the continuous increase in adsorption time, the increase rate of rhodamine B adsorption amount decreases, and the adsorption amount tends to be stable after a certain time. This is because, in the initial stage of adsorption, there are many active adsorption sites, which can have enough space to contain organic pollutants. With the advance of time, their holding capacity gradually reaches the peak, and the change of adsorption amount turns gentle [7]. The time for kaolin to reach adsorption equilibrium is about 90 min, and the maximum adsorption quantity for rhodamine B is 5.10 mg/g. The equilibrium time of kaolin sodium bentonite is 180 min, and the maximum adsorption capacity of Rhodamine B is 6.57 mg/g. The time for kaolin-organic bentonite to reach the adsorption equilibrium is about 180 min, and the maximum adsorption quantity for rhodamine B is 6.90 mg/g. After adding bentonite to kaolin, the adsorption rate and maximum adsorption quantity for rhodamine B increase. Kaolin-organic bentonite has the fastest adsorption rate and the highest adsorption amount for rhodamine B. The reason is twofold: (1) the scaly lamellar structure of bentonite provides a channel for the adsorption and migration of rhodamine B; (2) the rich adsorption sites on the surface of organic bentonite improve the adsorption efficiency for rhodamine B [23,29].

### 3.3. Effect of Initial Concentration of Rhodamine B Solution on Removal of Rhodamine B by Different Adsorbents

**Figure 5:**
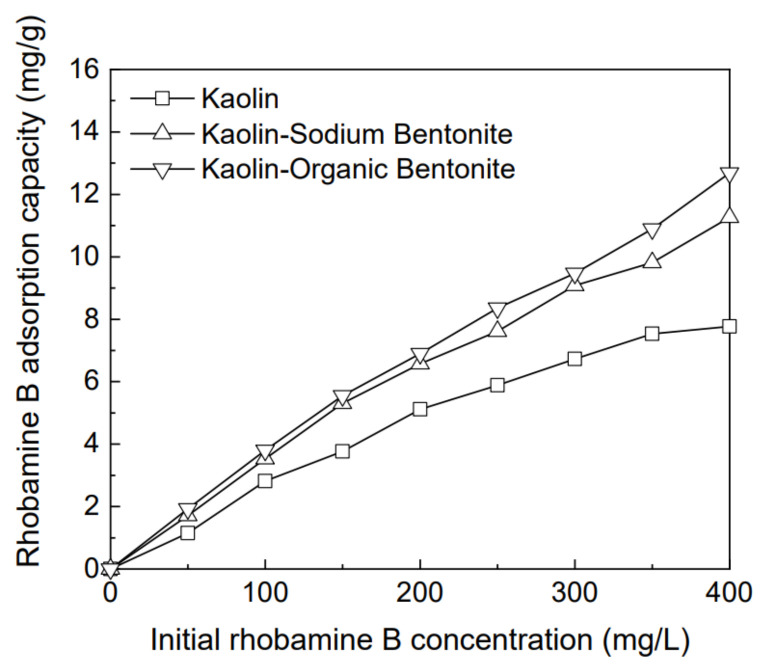
Effect of initial concentration of rhodamine B solution on removal of rhodamine B by different adsorbents.

Under the conditions of pH 9.1, temperature 25 °C, and adsorption time 360 min, the variation of the maximum adsorption quantity for rhodamine B, by different adsorbents with the initial concentration of the rhodamine B solution, is shown in Figure 5, where the initial concentration varies from 50 to 400 mg/L. It can be seen, from the figure, that the maximum adsorption quantity of kaolin for rhodamine B gradually stabilizes with the increase in the initial concentration of rhodamine B solution, while those of kaolin-sodium bentonite and kaolin-organic bentonite, for rhodamine B, always increase linearly with the increase in the initial concentration of rhodamine B solution. This implies that the adsorption amount for rhodamine B by the three adsorbents is positively correlated with the initial concentration of rhodamine B solution, and the adsorption amount of kaolin-sodium bentonite and kaolin-organic bentonite for rhodamine B still has the potential to increase. This may be related to the contact between the molecules of rhodamine B and the adsorption sites of the adsorbent. With the increase in the initial concentration of the rhodamine B solution, the contact between the molecules of rhodamine B and the available adsorption sites is promoted, and a higher number of rhodamine B molecules are adsorbed by the adsorbent [6,22]. When the initial concentration of rhodamine B was 400 mg/L, the adsorption capacities of kaolin, kaolin-sodium bentonite, and kaolin-organic bentonite were 7.76 mg/g (the corresponding rhodamine B removal rate was 48%), 11.26 mg/g (the corresponding rhodamine B removal rate was 70%), and 12.68 mg/g (the corresponding rhodamine B removal rate was 79%), respectively. Therefore, adding bentonite to kaolin can effectively improve its adsorption capacity for rhodamine B.

### 3.4. Effect of Temperature on Removal of Rhodamine B by Different Adsorbents

**Figure 6:**
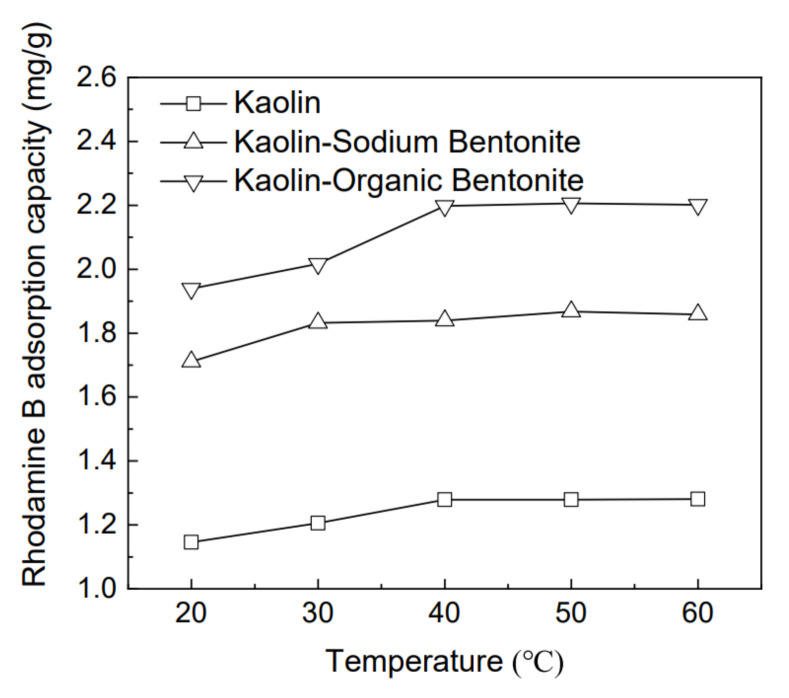
Effect of temperature on the removal of rhodamine B by different adsorbents.

Under the conditions of pH 9.1 and adsorption time 360 min, the adsorption capacity of the three adsorbents for 50 mg/L rhodamine B with temperature is shown in Figure 6, where the temperature range is 20–60 °C. The adsorption amount for rhodamine B by kaolin, kaolin-sodium bentonite, and kaolin-organic bentonite basically increases with the increase in temperature, but the increase rate of adsorption amount of the three adsorbents with temperature decreases at 40 °C, 30 °C, and 40 °C, respectively. With the further increase in temperature, the adsorption amount no longer increases significantly. The reason for this adsorption trend with the temperature variation may be that the increase in temperature promotes the diffusion rate of rhodamine B molecules in the adsorbent [30]. When the temperature was 60 °C, the three adsorbents reached the maximum adsorption quantity, and the corresponding adsorption capacities of rhodamine B were 1.28 mg/g, 1.85 mg/g, and 1.98 mg/g, respectively.

### 3.5. Effects of Adsorbent Dosage and pH on Removal of Rhodamine B by Kaolin-Organic Bentonite

**Figure 7:**
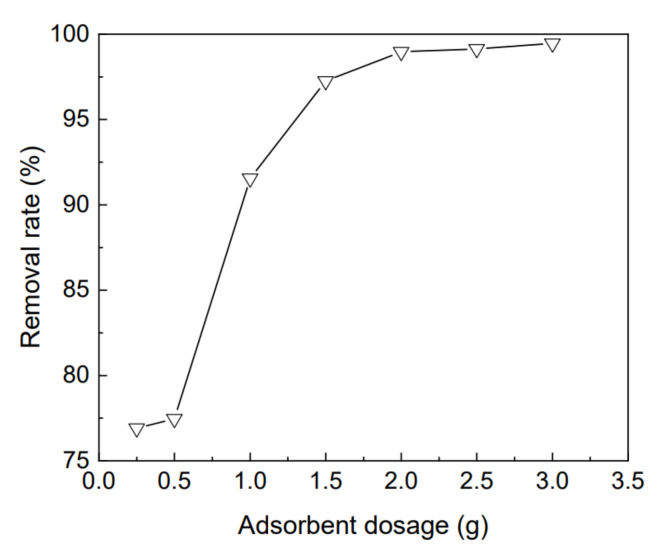
Variation of the removal rate of kaolin-organic bentonite for 50 mg/L rhodamine B with adsorbent dosage.

Under the conditions of pH 9.1, adsorption time of 360 min, and temperature of 25 °C, the removal rate for 50 mg/L rhodamine B, with different kaolin-organic bentonite dosages, is shown in Figure 7, where the dosage of kaolin-organic bentonite varies from 0.25 g to 3 g. When this dosage gradually increases from 0.25 g to 2.0 g, the removal rate for rhodamine B increases from 76% to 98%, displaying significant improvement. This is mainly due to the increase in adsorbent dosage, which increases the total surface area of the adsorbent and provides more adsorption sites. When the dosage of kaolin-organic bentonite continues to increase from 2.0 g to 3.0 g, its adsorption capacity for rhodamine B does not increase significantly, which may be related to the limited concentration of rhodamine B and the formation of an agglomerate in clay minerals [6].

**Figure 8:**
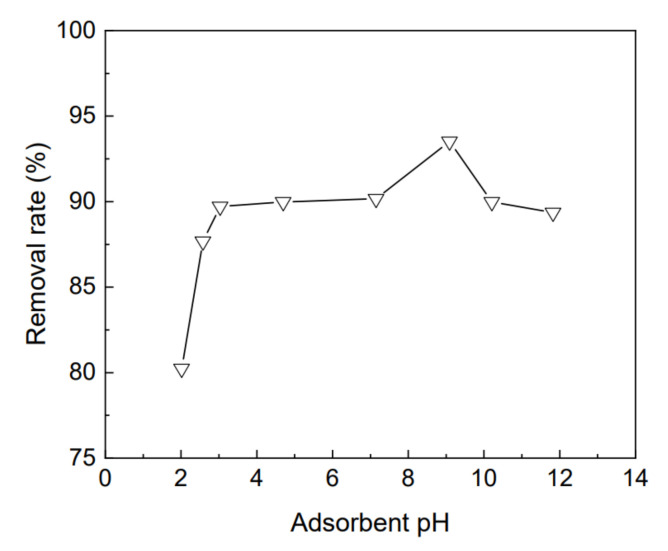
Variation of the removal rate of kaolin-organic bentonite, for 50 mg/L rhodamine B with adsorbent pH.

Under the conditions of adsorption time 360 min, temperature 25 °C, and adsorbent dosage 0.25 g, the variation of the removal rate of kaolin-organic bentonite, for 50 mg/L rhodamine B with pH value, is shown in Figure 8, where the pH value varies from 2.0 to 11.8. The results show that the adsorption for rhodamine B, by kaolin-organic bentonite, is closely related to pH [22,31]. When the pH value increases from 2.0 to 11.8, the adsorption capacity of kaolin-organic bentonite, for rhodamine B, first increases to the maximum value and then decreases. When the pH value was 2.0, the adsorption capacity was the lowest, and the removal rate was 80%. When the pH value was 9.1, the adsorption capacity reached the highest, and the removal rate for rhodamine B was 93%.

### 3.6. Adsorption Isotherm

The adsorption isotherm is of great significance to describe the interaction between adsorbate and adsorbent, as well as the adsorbent’s adsorption capacity. Freundlich and Langmuir models were used to fit the adsorption isotherms of the three adsorbents for different initial concentrations of rhodamine B (50–400 mg/L). Langmuir and Freundlich adsorption isotherm equations are shown in Equations (3) and (4), respectively [32]:

(3) $$\frac{C_{e}}{q_{e}}=\frac{1}{q_{\max}K_{L}}+\frac{C_{e}}{q_{\max}}$$

(4) $$\ln q_{e}=\ln K_{F}+\frac{\ln C_{e}}{n}$$

where *q_e_* is the adsorption capacity of the adsorbent when the adsorption reaches equilibrium (mg/g); *C_e_* is the concentration of rhodamine B after adsorption (mg/L); *q*_max_ is the maximum adsorption quantity (mg/g); *K_L_* is the Langmuir adsorption constant (L/mg); *K_F_* (L/g) and *n* are the Freundlich adsorption constants. Freundlich equation is used to describe the heterogeneous system and reversible adsorption, which is not limited to the formation of the monolayer. Langmuir adsorption isotherm assumes that the adsorption occurs at a specific homogeneous position in the adsorbent, which is most suitable for monolayer adsorption [23].

**Figure 9:**
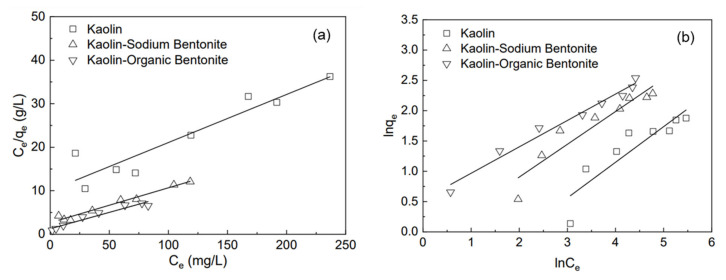
Adsorption isotherms of rhodamine B by different adsorbents: (**a**) Langmuir equation, (**b**) Freundlich equation.

Langmuir (Figure 9a) and Freundlich (Figure 9b) models were used to fit the test results, and the corresponding constants were calculated according to the intercept and slope. The relevant parameters are shown in Table 2. It can be seen from Table 2 that, for kaolin and kaolin-sodium bentonite, the correlation coefficient (R^2^) of the Langmuir model is significantly higher than that of the Freundlich model, while for kaolin-organic bentonite, the correlation coefficient of the Langmuir model is lower than that of the Freundlich model. Therefore, the Langmuir model is more suitable to describe the adsorption of rhodamine B by kaolin and kaolin-sodium bentonite, while the Freundlich model is more preferable to describe the adsorption of rhodamine B by kaolin-organic bentonite.

### 3.7. Adsorption Kinetics

The results were analyzed using pseudo-first order and pseudo-second order kinetic models to determine the adsorption characteristics of the adsorbent for rhodamine B. The adopted pseudo-first order and pseudo-second order kinetic models are shown in Equations (5) and (6), respectively [33]:

(5) $$\ln(q_{e}-q_{t})=\ln q_{e}-tK_{1}$$

(6) $$\frac{t}{q_{t}}=\frac{1}{q_{e}^{2}K_{2}}+\frac{t}{q_{e}}$$

where *K*_1_ is the pseudo-first order adsorption rate constant (min^−1^), *K*_2_ is the quasi-second order adsorption rate constant (g·mg^−1^ min^−1^), and *q_e_* and *q_t_* are the unit adsorption quantity (mg/g) for rhodamine B at adsorption equilibrium and time *t*, respectively.

**Figure 10:**
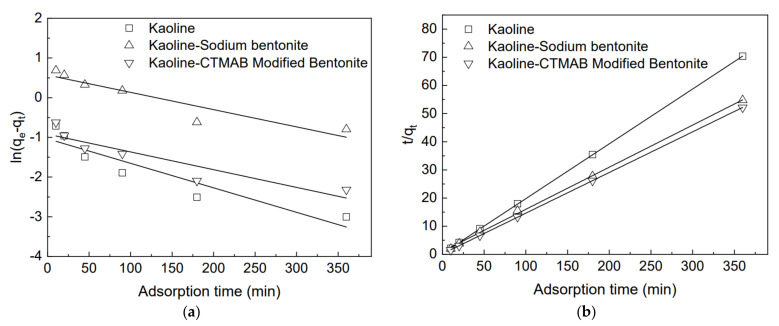
Adsorption kinetics of rhodamine B by different adsorbents: (**a**) pseudo-first order kinetic model; (**b**) pseudo-second order kinetic model.

The fitting results of adsorption kinetics of rhodamine B by different adsorbents are shown in Figure 10, and the relevant parameters of fitting are shown in Table 3. It can be seen that some correlation coefficients of the pseudo-first order kinetic model are lower than 0.90 and always lower than those of the pseudo-second order kinetic model, while the correlation coefficient of the pseudo-second order kinetic model is always higher than 0.98. The correlation coefficient of the pseudo-second order kinetic model is closer to 1 than that of the pseudo-first order kinetic model, which indicates that the adsorption kinetic characteristics of rhodamine B, by kaolin, kaolin-sodium bentonite, and kaolin-organic bentonite, are suitable to be described by the pseudo-second order kinetic model. This model is based on the adsorption capacity of the solid phase and considers that the driving force of the adsorption is the difference between the solid phase concentration at any time and at equilibrium [22].

### 3.8. Thermodynamic Study

**Figure 11:**
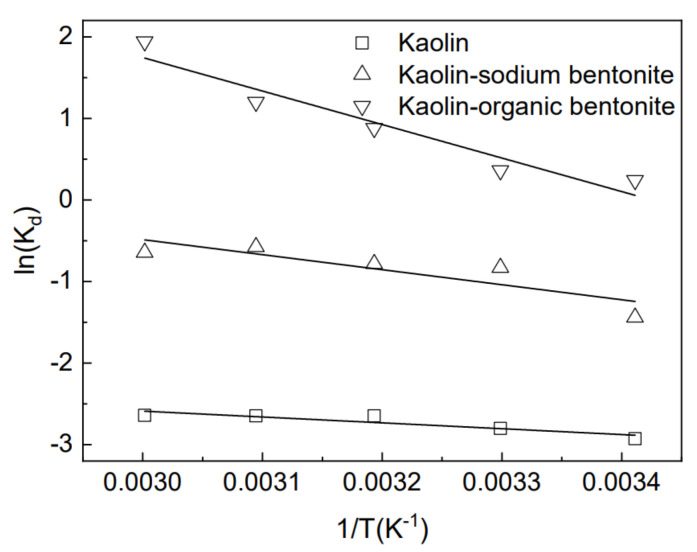
Plots of ln K_d_ versus 1/T for the three adsorbents.

The thermodynamic parameters can be obtained on the basis of the following equations [23]:

(7) $$\Delta G^{0}=-RT\ln K_{d}$$

(8) $$\Delta G^{0}=\Delta H^{0}-T\Delta S^{0}$$

(9) $$\ln(K_{d})=\frac{\Delta S^{0}}{R}-\frac{\Delta H^{0}}{RT}$$

where ΔG° is the standard adsorption free energy change (kJ/mol), ΔH° is the standard adsorption enthalpy change (kJ/mol), ΔS° is the standard adsorption entropy change (kJ/(mol K)), R is universal gas constant (8.324 kJ/mol), T is absolute temperature (*K*), and *K_d_* is the equilibrium constant. The values of ΔH° and ΔS° were obtained from the slope and intercept of the plots of ln(*K_d_*) against 1/T (Figure 11).

Table 4 shows the thermodynamic parameters of Rhodamine B adsorption by three adsorbents at different temperatures. Δh° is a positive value, indicating the endothermic nature of the interaction. The increase in temperature was conducive to the absorption of dyes [6]. ΔS° is a positive value, indicating that randomness of the solid/solution interface was enhanced during the adsorption of dye onto the adsorbents. Δg° is a negative value, indicating that the adsorption process was spontaneous and feasible for the adsorbent [23].

## 4. Conclusions

This work proves that adding 10% sodium bentonite or organic bentonite to kaolin can effectively enhance its adsorption capacity for rhodamine B. The adsorption properties of the three adsorbents for rhodamine B are positively correlated with temperature, and the most suitable pH value is about 9.1. In the initial stage of adsorption tests (the first 10 min), the adsorption amount for rhodamine B by kaolin, kaolin-sodium bentonite, and kaolin-organic bentonite increases rapidly, and then, the increase rate of the adsorption amount decreases rapidly, and the adsorption quantity gradually stabilizes. 

Under the conditions of temperature 25 °C, adsorption time 360 min and pH 9.1, with the gradual increase in the initial concentration of rhodamine B from 50 mg/L to 400 mg/L, the maximum adsorption quantity of kaolin for rhodamine B gradually increases and stabilizes, while the maximum adsorption quantity of kaolin-sodium bentonite and kaolin-organic bentonite shows a linear positive correlation with the initial concentration of rhodamine B. The maximum adsorption quantities for rhodamine B by kaolin, kaolin-sodium bentonite, and kaolin-organic bentonite were 7.76 mg/g (the corresponding removal efficiency was 48%), 11.26 mg/g (the corresponding removal efficiency was 70%), and 12.68 mg/g (the corresponding removal efficiency was 79%), respectively. 

The Langmuir isotherm model is suitable to describe the adsorption of rhodamine B by kaolin and kaolin-sodium bentonite, while it is preferable to use the Freundlich isotherm model in the case of kaolin-organic bentonite. The adsorption kinetic characteristics of rhodamine B by the three adsorbents are suitable to be described with the pseudo-second order kinetic model. This study shows that the use of kaolin and organic bentonite can design efficient adsorbents for organic pollutants similar to rhodamine B.

## Figures and Tables

**Table 1:** Batch adsorption test scheme.

Adsorbent	Rhodamine B Solution Concentration (mg/L)	Adsorption Time (min)	Adsorption Temperature (℃)	pH Value	Adsorbent Dosage (g)
kaolin, kaolin-sodium bentonite, kaolin-organic bentonite	50, 100, 150, 200, 250, 300, 350, 400	360	25	9.1	0.25
kaolin, kaolin-sodium bentonite, kaolin-organic bentonite	50, 100, 150, 200	10, 20, 45, 90, 180, 360	25	9.1	0.25
kaolin, kaolin-sodium bentonite, kaolin-organic bentonite	50	360	20, 30, 40, 50, 60	9.1	0.25
kaolin-organic bentonite	50	360	25	2.0, 2.6, 3.0, 4.7, 7.2, 9.1, 10.2, 11.8	0.25
kaolin-organic bentonite	50	360	25	9.1	0.25, 0.5, 1.0, 1.5, 2.0, 2.5, 3.0

**Table 2:** Adsorption isotherm parameters.

Adsorption Isotherm Equation	Parameter	Kaolin	Kaolin-Sodium Bentonite	Kaolin-Organic Bentonite
Langmuir	*q_m_* (mg/g)	8.23477	11.96847	13.31723
	*K_L_* (L/mg)	0.01522	0.03542	0.05512
	R^2^	0.88	0.97452	0.945
Freundlich	*K_F_* (L/g)	0.60793	1.30457	1.77013
	*n*	2.26767	2.32162	2.35557
	R^2^	0.77507	0.87509	0.97686

**Table 3:** Kinetic fitting of rhodamine B adsorption by different adsorbents.

Adsorbent Concentration (mg/L)		50		
Adsorbent Type		Kaolin	Kaolin-Sodium Bentonite	Kaolin-Organic Bentonite
Pseudo-first order kinetic equation	*q_e_* (mg/g)	5.1152	6.57354	6.90133
	*K*_1_	0.26364	0.15821	0.30208
	R^2^	0.83987	0.84537	0.80484
Pseudo-second order kinetic equation	*q_e_* (mg/g)	5.1649	7.02359	6.82869
	*K*_2_	0.20192	0.04874	0.22165
	R^2^	0.99994	0.98391	0.99967

**Table 4:** Thermodynamic parameters for the adsorption of rhodamine B by the three adsorbents.

Temperature/(℃)		20	30	40	50	60
Kaolin	Δ*H*^0^/(kJ mol^−1)^	5.780				
	Δ*S*^0^/(kJ mol^−1^K^−1^)	0.053				
	Δ*G*^0^/(kJ mol^−1^)	−9.700	−10.340	−11.090	−11.450	−11.810
	R^2^	0.970				
Kaolin-sodium bentonite	Δ*H*^0^/(kJ mol^−1)^	4.990				
	Δ*S*^0^/(kJ mol^−1^K^−1^)	0.053				
	Δ*G*^0^/(kJ mol^−1^)	−10.680	−11.400	−12.040	−12.460	−12.840
	R^2^	0.872				
Kaolin-organic bentonite	Δ*H*^0^/(kJ mol^−1)^	4.060				
	Δ*S*^0^/(kJ mol^−1^K^−1^)	0.051				
	Δ*G*^0^/(kJ mol^−1^)	−10.980	−11.550	−12.210	−12.620	−13.030
	R^2^	0.906				

## Data Availability

The data that support the findings of this study are available from the corresponding author upon reasonable request.
